# Experimental Study of the Mechanical Properties of Full-Scale Rubber Bearings at 23 °C, 0 °C, and −20 °C

**DOI:** 10.3390/polym16070903

**Published:** 2024-03-25

**Authors:** Hui Pang, Tao Jiang, Junwu Dai, Yongqiang Yang, Wen Bai

**Affiliations:** 1 Key Laboratory of Earthquake Engineering and Engineering Vibration, Institute of Engineering Mechanics, China Earthquake Administration, Harbin 150080, China; panghui0615@163.com (H.P.); junwudai@126.com (J.D.); yangyongqiang@iem.ac.cn (Y.Y.); baiwen@iem.ac.cn (W.B.); 2 Key Laboratory of Earthquake Disaster Mitigation, Ministry of Emergency Management, Harbin 150080, China; 3 School of Civil Engineering & Transportation, Bei Hua University, Jilin 132013, China

**Keywords:** isolation rubber bearings, full scale, ambient temperature, correction methodologies, empirical fitting formulations

## Abstract

In this study, the effects of ambient temperature on the horizontal mechanical performance of isolated rubber bearings were investigated using high-speed reciprocating loading methods. A comprehensive series of 54 experimental trials are performed on the full-scale (900 mm-diameter) isolation rubber bearings, encompassing a range of temperatures (−20 °C, 0 °C, and 23 °C), shear pressures (50%, 100%, and 250%), and frequencies (0.20 Hz, 0.25 Hz, and 0.30 Hz). Because the compression-shear tests were conducted at high velocities and pressures (specifically, vertical compressive stress of 15 MPa), the equipment used in these tests was capable of generating substantial inertial and frictional forces. Appropriate correction methodologies for the precise determination of mechanical performance metrics for bearings are presented. Then, a comprehensive investigation of the effects of various loading conditions on the characteristic strength, post-yield stiffness, horizontal equivalent stiffness, and equivalent damping ratio of LRB900 (lead-core rubber bearings 900 mm-diameter) and LNR900 (linear natural rubber bearings 900 mm-diameter) is conducted. The empirical results show a discernible relationship between these characteristics and ambient temperature as the number of loading cycles increases, except for the equivalent damping ratio. Finally, empirical fitting formulations incorporating the influence of ambient temperature are presented for each performance indicator. These formulas are intended to assist designers in performing seismic design analyses by allowing them to take into consideration the effects of ambient temperature comprehensively.

## 1. Introduction

The utilization of base isolation technology, which is a pragmatic method for enhancing the seismic resistance of structures, has garnered extensive recognition among professionals in the field of engineering. Isolation rubber bearings have emerged as a popular choice for seismic devices due to their simple construction, reasonably consistent performance, and cost-effectiveness [1,2]. The aforementioned bearings consist of natural rubber bearings, lead-core rubber bearings, and high-damping rubber bearings. These bearings are commonly fabricated by interleaving layers of rubber with slender steel plates. Not only are they capable of supporting considerable vertical loads, but they also demonstrate notable horizontal deformability. The implementation of base isolation technology involves the integration of isolation devices into structures. This integration serves to prolong the natural vibration periods of the structures, improve damping characteristics, and ultimately reduce the adverse effects of earthquakes on the upper structure, building equipment, and the safety of contents [3]. As of December 2022, China has emerged as a prominent contributor to the worldwide construction landscape, with a substantial proportion of its existing or under-construction structures using seismic and isolation techniques. Buildings that are isolated, known for their commendable ability to withstand seismic forces, are widely utilized in regions prone to earthquakes for crucial structures such as hospitals, information centers, and hazardous material storage facilities. They are also frequently employed in significant infrastructure projects such as large-scale transportation hubs. Nevertheless, the rubber-bearing isolation system has encountered growing complexity in its application environment due to the ongoing progress in isolation technology. As a result, the impact of ambient temperature on the efficacy of the isolation system has increasingly become a central focus within the realms of engineering and academia.

Several recent studies [4,5,6,7] have revealed a noteworthy correlation between temperature and the behavior of rubber and its isolators. However, it is worth noting that existing design standards for isolated structures do not yet incorporate considerations related to temperature. Hence, in order to guarantee the safety of isolated structure designs and gain a comprehensive understanding of the temperature-related properties of the isolators, it is crucial to develop design methodologies that incorporate temperature effects. The performance of rubber isolators is influenced by temperature, primarily manifesting in phenomena such as low-temperature transient hardening, low-temperature crystalline hardening, and hysteresis heating. Currently, there is a significant focus on studying the temperature-related properties of these isolators, both domestically and internationally. This research primarily takes two forms: experimental investigations, which involve quasi-static tests and shake table tests, and theoretical analyses that examine the energy conversion occurring within the isolators.

Significant findings pertaining to the temperature-dependent performance of rubber isolators have been obtained in an experimental study. In their study, Yang et al. [8] performed quasi-static experiments on LRB500 across a temperature range of −40 °C to 23 °C. The results indicated a significant disparity in the mechanical performance metrics of LRB at low temperatures in comparison to room temperature. In a study conducted by Shi et al. [9], compression-shear tests were conducted on rubber bearings with a diameter of 400 mm under winter temperatures in Shenyang. The results indicated that low temperatures have a notable impact on both the vertical compression stiffness and the horizontal stiffness of the rubber bearings. The study conducted by Cheng et al. [10] investigated the influence of bearing temperature on the seismic response of isolated structures. The researchers observed that the effectiveness of isolation was improved when the temperature of the bearings increased within the typical operational limits. Pinarbasi et al. [11] conducted shear loading experiments on rectangular bridge bearings measuring 200 mm × 400 mm. The researchers investigated the shear performance of these bearings under various temperatures and strain levels. The findings revealed that the stiffness of the bearings at low temperatures was approximately twice as high as that at normal temperatures. The seismic response of isolated structures in cold regions was examined by Nakano et al. [12]. Their study revealed significant alterations in the response, which were attributed to the impact of temperature on the performance of bearings. Liu et al. [13] conducted research on the temperature-dependent behavior of mechanical parameters in rubber bearings. They put forth temperature correction equations for post-yield stiffness and characteristic strength. In their study, Li et al. [14] conducted experiments on two types of lead-core rubber isolators, specifically those with 400 and 600 types. These studies were conducted throughout a temperature range of −40 to 40 °C. Through nonlinear regression analysis, the researchers proposed an approximation formula that captures the influence of temperature on the mechanical performance of the isolators. The temperature-dependent characteristics of rubber bearings with diameters of 600 mm and 1200 mm were investigated by Zhuang et al. [15,16]. The study focused on the effects of temperature on the vertical and horizontal stiffness as well as the characteristic strength of these bearings. Hu et al. [17] generated regression curves for mechanical performance parameters based on the test findings. They subsequently conducted an analysis to examine the impact of different temperatures on the seismic response of isolated bridges. Liu et al. [18] conducted an analysis of the seismic response of high-rise isolated structures across a temperature range of −40 °C to 40 °C. Their research highlights the notable impact of temperature performance on the seismic reaction within the context of isolation design. Zhang and Li [19] conducted a comprehensive investigation into the influence of ambient temperature on the mechanical properties of scaled-down bearings. The study involved a series of variable-temperature pressure-shear experiments conducted within a temperature range of −30 °C to 20 °C. The evaluation of the effects of temperature on important mechanical properties, such as horizontal equivalent stiffness, characteristic strength, and post-yield stiffness, has been the focus of numerous studies. Yasar et al. [20] proposed a theoretical equation for predicting the mechanical performance variability of a lead rubber bearing (LRB) in low-temperature conditions. They successfully determined the ranges of post-yield stiffness and characteristic strength fluctuations for LRB exposed to varying durations of low-temperature conditions.

Many remarkable advances have been made in theoretical research on the dynamics and characteristics of seismic isolators. According to the research of Kalpakidis and Constantinou [21,22], it was hypothesized that the dissipation of energy in LRB occurs mainly through the conversion of seismic input energy into thermal energy within the lead core and rubber. This hypothesis was founded on the principle of energy conservation. Constantinou et al. [23] extensively studied the mechanical properties of LRB. Their study encompassed several factors, such as aging and temperature, and used a combination of theoretical studies, numerical simulations, and experimental research. Correction coefficients were introduced to account for these specific variables. These adjustment factors were subsequently incorporated into the AASHTO seismic design guidelines [24]. Constantinou et al. [25] proposed an improved bilinear hysteresis model and investigated its dynamic response. The simulation outcomes of this model exhibited a high level of concordance with empirical observations, thereby confirming the precision of the aforementioned theory and the improved model. The study conducted by Ozdemir et al. [26,27,28,29] added to the existing body of knowledge by examining the effect of temperature fluctuations in the lead core of bearings caused by seismic activity under various site conditions, on the seismic behavior of isolated bridges. The researchers conducted a comparative analysis of the temperature variations in the lead core when subjected to unidirectional and bidirectional seismic coupling. They also examined the resulting effect on the structural response. In addition, they proposed a parameter correction formula for the equivalent linearization method of the LRB that takes temperature changes in the lead core into account. Deng et al. [30] used a nonlinear dynamic analysis to investigate the negative effects of low temperatures on the seismic behavior of highway overpasses that contain LRBs. Their findings revealed that, under low-temperature conditions, the isolation effectiveness of LRB, which was originally designed for use at room temperature, is significantly diminished. Miyamura et al. [31] introduced a thermomechanical linked finite element method to analyze laminated high-damping rubber bearings (HDRBs). The study conducted by Fu et al. [32] investigated the seismic performance of isolated bridges by taking the temperature dependence of LRB into account. They concluded that the heating effect caused by seismic loads on the lead core is significant and should be taken into account in the design of bridge isolation systems. Shi et al. [33] investigated the effect of regional temperature uncertainty on the seismic vulnerability of LRB-isolated bridges. Integration of earthquake vulnerability analysis with a backpropagation (BP) neural network allowed them to accomplish this. Tan et al. [34] presented a simplified temperature-coupled hysteresis model for high-damping seismic bearings. The objective of their study was to investigate the influence of low temperatures on the hysteresis performance of these bearings.

Extensive research has been conducted both domestically and internationally to investigate the mechanical characteristics of seismic isolators at different temperatures. This research found that the mechanical performance of lead-core rubber seismic isolators is substantially affected by temperature, especially when exposed to low-temperature conditions. However, the current body of research on the high- and low-temperature performance of seismic rubber materials is inadequate, primarily due to constraints in experimental methodologies and controlled environments. Currently, there is a paucity of comprehensive research on the positive and negative effects of internal temperature increases on lead cores and rubber materials caused by dynamic loading during large earthquakes with protracted periods and durations. The Chinese standard, known as “Rubber Bearings Part 1: Test Methods for Seismic Rubber Bearings (GB/T 20688.1-2007)” [35], stipulates the temperature’s effect on rubber bearings. This standard requires temperature testing for seismic rubber bearings. However, the current recommendations only require tests to be conducted at a single frequency and strain, resulting in inadequate coverage of the mechanical characteristics of seismic rubber isolators under low-temperature conditions. There is insufficient research on a comprehensive analysis of the effects of low temperatures on base-isolated structures.

In comparison to other fields of study, the examination of temperature coupling effects in rubber isolation engineering and their implications for seismic safety in affluent nations like the United States and Japan remains understudied. Although there have been advancements in the field, current research mostly focuses on small-scale experimental investigations of seismic rubber isolators. These studies often involve slow loading speeds that unfortunately leave some important questions unresolved. Considering the rapid development of seismic engineering in regions characterized by a cold climate and high-intensity conditions, a sequence of compression-shear tests on rubber isolators (specifically, LRB900 and LNR900) was conducted using a high-speed compression-shear testing apparatus in the present study. In the experiments, different temperatures (−20 °C, 0 °C, and 23 °C), frequencies (0.20 Hz, 0.25 Hz, and 0.30 Hz), and shear stresses (50%, 100%, and 250%) were investigated to determine the effect of ambient temperature on the mechanical characteristics of seismic rubber isolators. This research presents a correction approach for precisely determining the performance indicators of bearings during high-speed, high-pressure tests conducted under a vertical stress of 15 MPa. The method accounts for the significant inertial and frictional forces generated by the equipment during testing. In addition, a comprehensive study was conducted to analyze the mechanical performance indicators of rubber isolators as a function of temperature, based on the experimental results obtained. These indicators include characteristic strength, post-yield stiffness, horizontal equivalent stiffness, and equivalent damping ratio. In addition, this research provides a thorough investigation of the adjustment factors that account for the influence of ambient temperatures on the aforementioned features, which can serve as an important reference source for design professionals.

## 2. Test Introduction

### 2.1. The Information of the Specimen

Six different types of rubber bearings are examined in this article: three natural rubber bearings (LNR900-1, LNR900-2, and LNR900-3) and three lead rubber bearings (LRB900-1, LRB900-2, and LRB900-3). Table 1 presents a complete overview of the precise measurements and dimensions of the rubber bearings.

### 2.2. The Measurement of the Ambient Temperature

**Figure 1:**
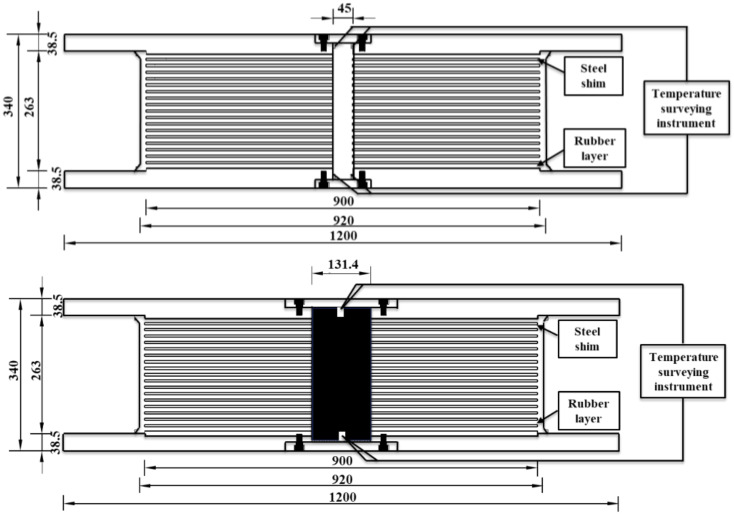
Configuration features of the bearing and the location of the thermocouple (unit: mm).

This section first explains the method used to cool the specimens to allow high-speed compression-shear experiments to be performed on rubber bearings under ambient temperatures of −20 °C and 0 °C. Before beginning the cooling procedure, the team affixed four sets of thermocouples to both the lead core and the inner wall of the central hole. The thermocouples were used to monitor and document any temperature variations within the bearing. Figure 1 shows the arrangement of the bearing and the positioning of the thermocouple, which was positioned 5 cm from the inner surface of the cover plate.

**Figure 2:**
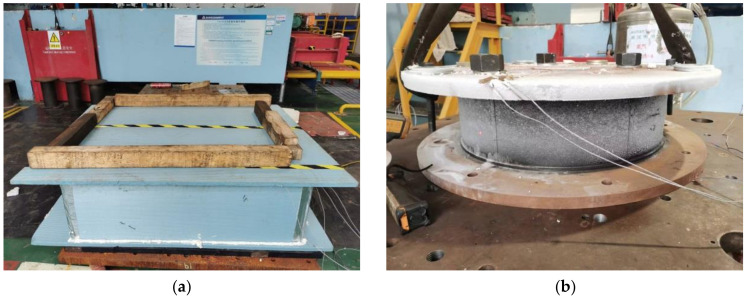
Cooling process of specimen: (**a**) Thermal insulation device; (**b**) Cooling specimen.

During the experiment, dry ice was continuously supplied without interruption to lower the temperature of the rubber bearing. As shown in Figure 2, the rubber bearing was initially placed in an incubator that was continuously supplied with dry ice. Using the upper and lower cover plates, this configuration facilitated the efficient transfer of low temperatures to the bearing. To adhere to the test standards, it is imperative that the thermocouple reading remain marginally lower than the required test temperature. This adjustment accounted for the rise in temperature caused by the installation technique used for the specimen. It is advisable to maintain this specific temperature range for a period of one to two hours, to allow the rubber bearing sufficient time to attain thermal equilibrium. The sample was then efficiently transferred to the testing apparatus, allowing the compression-shear test to commence immediately.

### 2.3. Test Loading Scheme

**Figure 3:**
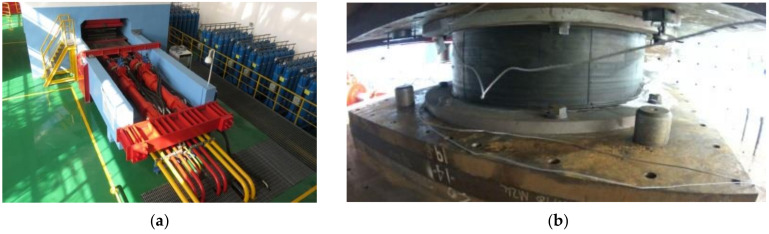
Compression-shear test of specimen: (**a**) Multifunctional apparatus; (**b**) Installation of the specimen.

As shown in Figure 3, the multifunctional apparatus used in this experiment had a vertical maximum load capacity of 75,000 kN, a vertical stroke of 120 mm, a horizontal maximum load capacity of 6000 kN, a horizontal stroke of 600 mm, and a horizontal maximum loading velocity of 1000 mm/s.

To determine the effects of various factors on the mechanical properties of rubber bearings, a series of tests were conducted under 54 distinct conditions. These conditions were designed in accordance with the national standard ‘Rubber Isolators Part 1: Test Methods for Seismic Rubber Isolators’ (GB/T 20688.1-2007) [35]. The factors investigated were rubber isolator type, shear deformation, loading frequency, and ambient temperature. The loading conditions have been summarized and are listed in Table 2. The vertical load is the load supported by the rubber bearings when subjected to a conventional fortification limit of 15 MPa. The horizontal loading frequencies are defined as 0.20 Hz, 0.25 Hz, and 0.30 Hz, with peak velocities ranging from 107.4 to 805.8 mm/s. In accordance with the applicable provisions outlined in the ‘Code for Seismic Design of Buildings’ GB 50011-2010 [36] and the ‘Standard for Seismic Design of Building Isolation’ GB/T 51408-2021 [37], the shear strain amplitudes were set at levels of 50%, 100%, and 250% to account for the effects of minor, moderate, and major seismic events, respectively. In addition, temperature-based experimental procedures were conducted at three specific temperatures: −20 °C, 0 °C, and 23 °C, in accordance with the guidelines outlined in the standards ‘Rubber Isolators Part 2: Bridge Seismic Rubber Isolators’ GB/T 20688.2-2006 [38] and ‘Rubber Isolators Part 3: Building Seismic Rubber Isolators’ GB 20688.3-2006 [39]. Before initiating the second set of tests, the isolators were given a minimum of 24 h of rest between each test to minimize the negative effects of temperature fluctuations on the subsequent tests.

**Figure 4:**
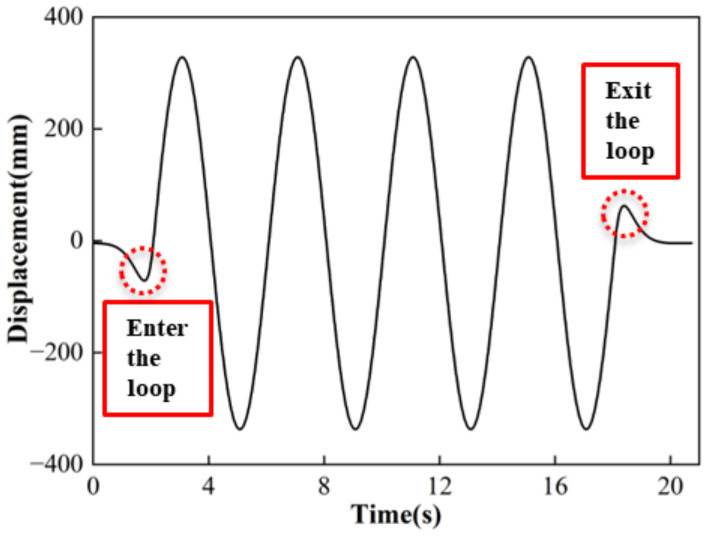
Sine loading waveform.

The experimental system used a sine wave to control the horizontal movement. To reduce the impact of the inertial force exerted by the equipment at the beginning and end stages, the current study incorporated the entrance and exit cycles into the loading waveform, as depicted in Figure 4.

## 3. Experimental Results

In the ongoing experiment, the apparatus was subjected to high-velocity, high-pressure, and enormous deformation loads. Consequently, substantial inertial and frictional forces were generated within the moving components of the device, including the test specimen. These forces have a substantial effect on the precision and dependability of the experimental findings. Therefore, the subsequent sections of this paper provide a comprehensive elucidation of the modifications made to resolve the inertial and frictional forces in the apparatus.

### 3.1. Corrections Made to the Test Data

#### 3.1.1. Correction of Equipment Inertial Force

**Figure 5:**
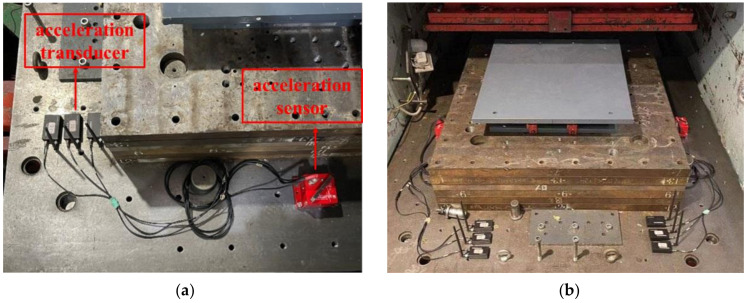
Mounting spherical slide bearing and accelerometers: (**a**) Installation of accelerometers; (**b**) Installation of spherical slide bearing.

To quantify the inertial forces exerted by the moving components, the acceleration-measuring equipment was positioned at the center of the working platform (Figure 5a). In addition, a moving platform was fitted with spherical sliding bearings similar to those found in the loading system (Figure 5b). The measurement of the spherical sliding bearing requires consideration of both the internal spherical sliding bearings of the equipment and the hysteresis curve of the restoring force of the specimen. These elements were characterized by rectangular hysteresis loops, which were the result of Coulomb friction. During the measurement process, the horizontal force exerted by the loading system can be understood as a combination of the inertial forces generated by the moving components and a larger rectangular hysteresis loop.

**Figure 6:**
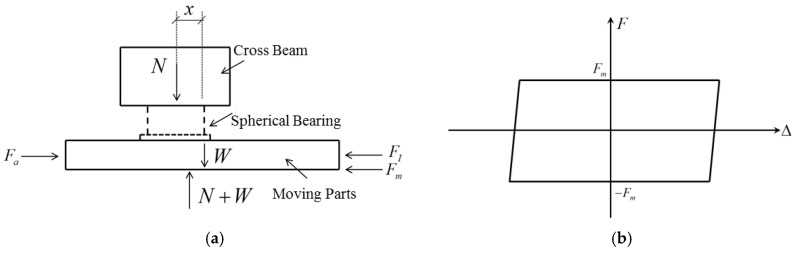
Force diagram of the test apparatus: (**a**) Force diagram of the moving parts; (**b**) Internal friction of system and restoring force of spherical slide bearing.

Figure 6a shows a simplified force diagram of the complete loading equipment. The horizontal forces at play consist of the tractive force *F_a_*, which is supplied by the horizontal actuator, the inertial force *F_I_* resulting from the motion of the components, and the combined restoring force *F_m_* of the specimen, along with the internal friction within the system. In the vertical plane, the forces present include the reaction force *N* exerted by the reaction crossbeam, self-weight W of the moving components (including the specimen), and vertical force *N* + *W* exerted by the vertical actuator that sustains the moving platform. The combination of the internal friction inside the system and the restoring force exerted by the spherical sliding bearings produced a hysteresis loop, as depicted in Figure 6b. This loop is comparable to a parallelogram enclosed by Coulomb friction. It is important to note that when the specimen was moving horizontally, the first-order derivative of the friction force Fm with respect to deformation was equal to zero.

For the simplified force system, an equation representing the equilibrium of forces in the horizontal direction is provided.

(1) $$F_{a}=F_{m}+F_{I}$$

(2) $$F_{m}=F_{a}-F_{I}$$

Using the sinusoidal displacement loading technique in the experiment, the displacement at any given time is represented by the following equation:

(3) x=Asinωt

The equation is defined as follows: *A* represents the amplitude of displacement, *ω* symbolizes the circular frequency of loading, and *t* specifies the time of loading.

Currently, the relationship between acceleration and displacement is expressed analytically as follows:

(4) $$a=-\omega^{2}x$$

Thus, the correlation between the inertial force generated by the mobile components and the displacement is as follows:

(5) $$F_{I}=ma=m_{h}(-\omega^{2}x)$$

The equation incorporates the variable *m_h_*, which represents the cumulative mass of various moving elements. These elements included the piston cylinders of the four horizontal actuators, the mobile platform, the tooling plate, the mobile cart, and the test samples under examination.

Given that the first derivative of the function *F_m_* with respect to the displacement variable *x* is equal to zero, we can proceed by differentiating both sides of Equation (2), with respect to *x*, resulting in the following equation:

(6) $$0=\frac{dF_{a}}{dx}-\frac{dF_{I}}{dx}$$

(7) $$\frac{dF_{I}}{dx}=-m_{h}\omega^{2}$$

To perform a sinusoidal displacement-controlled loading test on a spherical sliding bearing, the hysteresis loop of the actuator in relation to the horizontal traction force as a function of displacement must be determined. Additionally, it is important to ascertain the hysteresis loop of the accelerometer in relation to the acceleration as a function of displacement. The masses of the moving components can be determined by combining Equations (4) and (7).

(8) $$k_{x}=\frac{da}{dx}=-\omega^{2}$$

(9) $$k_{y}=\frac{dF_{a}}{dx}=\frac{dF_{I}}{dx}=-m_{h}\omega^{2}$$

The variables *k_x_* and *k_y_* in the above equations represent the slopes of the accelerometer and horizontal actuator readings, respectively, with relation to displacement *x.*

**Figure 7:**
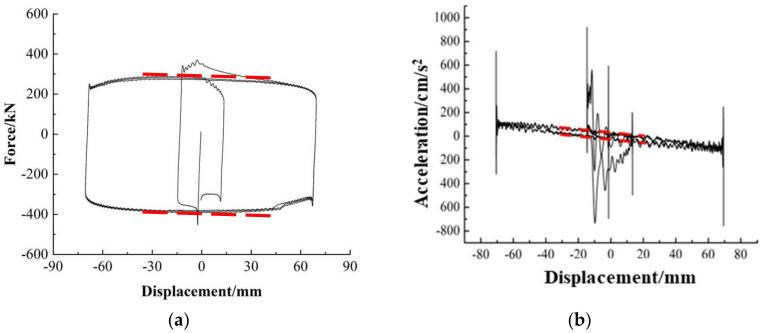
Correction process of equipment inertia force: (**a**) The hysteresis loop of spherical sliding bearing; (**b**) The hysteresis loop of accelerometers.

In Figure 7a, the hysteresis loop of the horizontal force exerted by the horizontal actuator is presented as a function of displacement. The red dashed line in the figure serves as a schematic representation for determining *k_y_*. Similarly, Figure 7b shows the hysteresis loop of acceleration as a function of displacement. The red dashed line in this figure served as the schematic line for determining *k_x_*. To mitigate the influence of changes in displacement direction on data oscillations, the values of *k_x_* and *k_y_* were determined by sampling data within the range of ±A/2. The mass of the moving component *m_h_* can be determined by dividing *k_y_* by *k_x_*. Once the mass of the moving components has been determined, the inertial force *F_I_* can be computed using the data obtained from the accelerometer.

#### 3.1.2. Correction of Equipment Friction Force

After determining and establishing the inertial force exerted by the moving components within the loading system, it was possible to analyze the restoring force of the spherical sliding bearing to estimate the internal frictional force present within the loading apparatus. The prescribed procedure consists of the following sequential steps.

**Figure 8:**
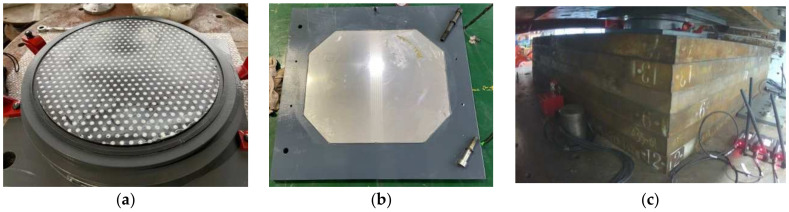
Compression-shear test of the spherical slide bearing: (**a**) Smearing silicone grease on the sliding contact surface; (**b**) Upper sliding contact surface; (**c**) The installation diagram of the spherical sliding bearing.

**Figure 9:**
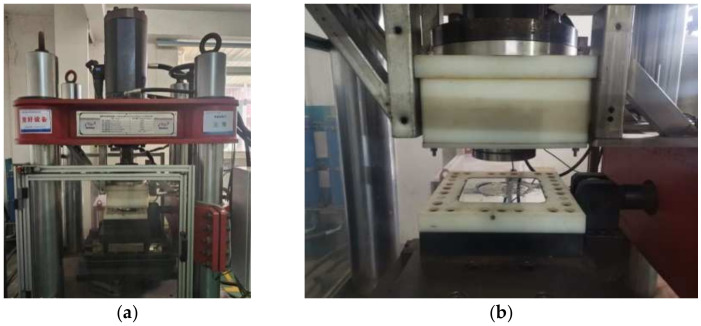
SMTM testing machine: (**a**) Hydraulic loading system; (**b**) Installation of friction materials.

**Figure 10:**
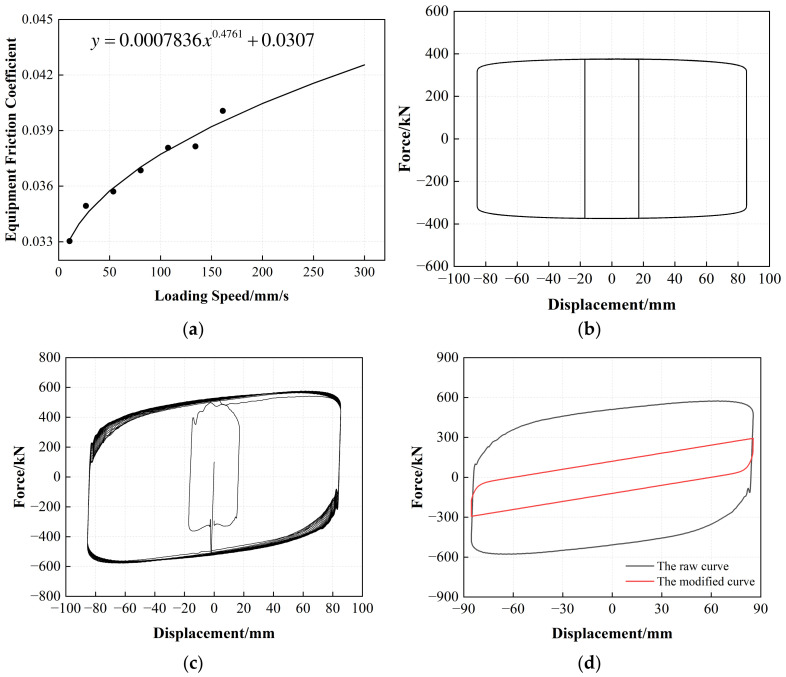
The correction process of the internal friction forces: (**a**) The correlation curve of the equipment friction coefficient and loading speed; (**b**) The hysteresis loop of equipment internal friction; (**c**) The hysteresis curve of LRB900 after deducting inertia force; (**d**) The shear-displacement hysteresis curves of LRB900.

(1) In the preliminary stage, a set of compressive shear tests were performed on spherical sliding bearing samples. In these tests, the displacement amplitudes and loading frequencies were varied, as shown in Figure 8. The purpose of these tests was to ensure that the experimental conditions included the required vertical compressive stress, displacement amplitude, and loading frequency for LRB900 and LNR900 in the official experiment.

(2) The high-polymer friction plates coated with silicone grease within the spherical sliding bearing samples were subjected to material tests using a small-scale testing machine, known as an SMTM (Figure 9). By conducting experiments in which a constant displacement amplitude is maintained while the loading frequency is adjusted, it is possible to quantify the frictional force and its corresponding friction coefficient under a Constant vertical compressive stress in accordance with the official experiment. This methodology enabled the characterization of the relationship between the variation of the friction coefficient and velocity.

(3) The recovery force of the bearing sample can be calculated by multiplying the friction coefficient of the spherical sliding bearing by the vertical pressure gauge measurement, denoted as *N*, of the device. The friction force of the system under specific operating conditions was determined by considering the output force *F_a_* of the horizontal actuator and the inertial force of the moving components. By dividing the frictional force by the sum of the normal force and the weight (*N* + *W*), one can determine the system’s friction coefficient and the associated peak velocity when subjected to this vertical force.

(4) The obtained data pairs between the internal friction coefficient of the spherical sliding bearing and peak loading velocity, as determined from the component test results at different displacement amplitudes and loading frequencies, were used to construct a relationship curve (Figure 10a) depicting the correlation between the loading velocity and the friction coefficient of the system. Based on this, the hysteresis loops of the friction coefficient and the loading velocity under different displacement amplitude conditions were calculated (Figure 10b).

(5) The hysteresis curve of LRB900, which eliminates the inertial force, can be determined by subtracting the inertial force of the moving components from the horizontal actuator output Fa (Figure 10c). Consequently, the reduction in system friction (Figure 10d) enabled the identification of the authentic horizontal hysteresis curve for the LRB900 sample.

**Figure 11:**
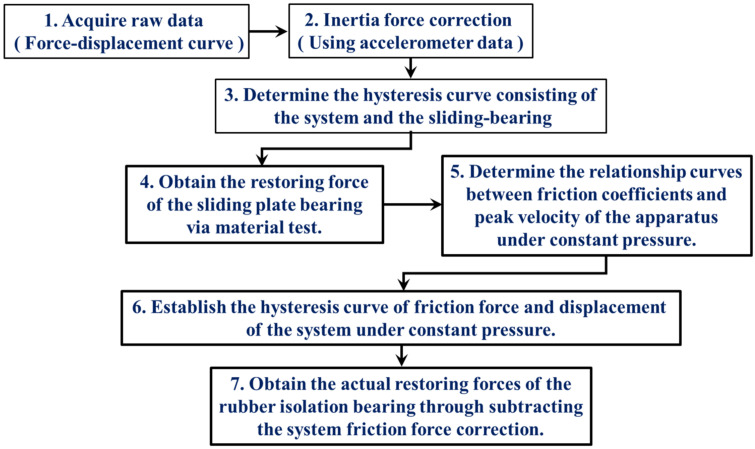
The flow chart of the internal forces and friction forces correction process.

It is crucial to note that during the experimental evaluation of the spherical sliding bearings, specifically during the testing procedures described in steps 1 and 3, the pressure gauge of the loading system was positioned at the lower section of the movable platform. Consequently, it was appropriate to describe the vertical force outcome as *N* + *W*. Before beginning the test, the researchers adjusted the platform’s vertical position to ensure that the bearing was aligned with the tooling plate. They then manually reset the vertical force reading to zero, thereby disregarding the self-weight (*W*) of the moving components. Consequently, the vertical force measured in the experiments represented the magnitude of the vertical force (*N*) exerted on the specimen. The frictional forces of LRB900 and LNR900 were determined using deductive reasoning. Multiplying the system friction coefficient by the sum of the normal force (*N*) and weight (*W*), where the weight varies based on the sample type and tooling plate thickness, yields the internal friction force resulting from the applied vertical force. Figure 11 illustrates the procedure for adjusting the inertial and frictional forces of the device.

To assess the veracity of the device techniques for correcting the inertial and friction forces, this investigation juxtaposed the low-speed test findings (loading frequency of 0.02 Hz) of specimens LRB900-1, LRB900-2, and LRB900-3 at room temperature, exhibiting shear strains of 50%, 100%, and 250%, respectively, with the official low-speed test results (loading frequency of 0.02 Hz) provided by the manufacturer. The results of the comparison are listed in Table 3, and the techniques used to determine the characteristic strength and post-yield stiffness can be found in [39].

Based on the information presented in Table 3, the adjusted test results for LRB900, which included both the characteristic strength and post-yield stiffness, were within 10% of the manufacturer-provided detection results. The present discovery provides robust support for the validity and dependability of the inertial and friction force correction techniques proposed in this study.

### 3.2. Relationship between the Number of Loading Cycles and Temperature Changes

#### 3.2.1. The Characteristic Strength of LRB900

**Figure 12:**
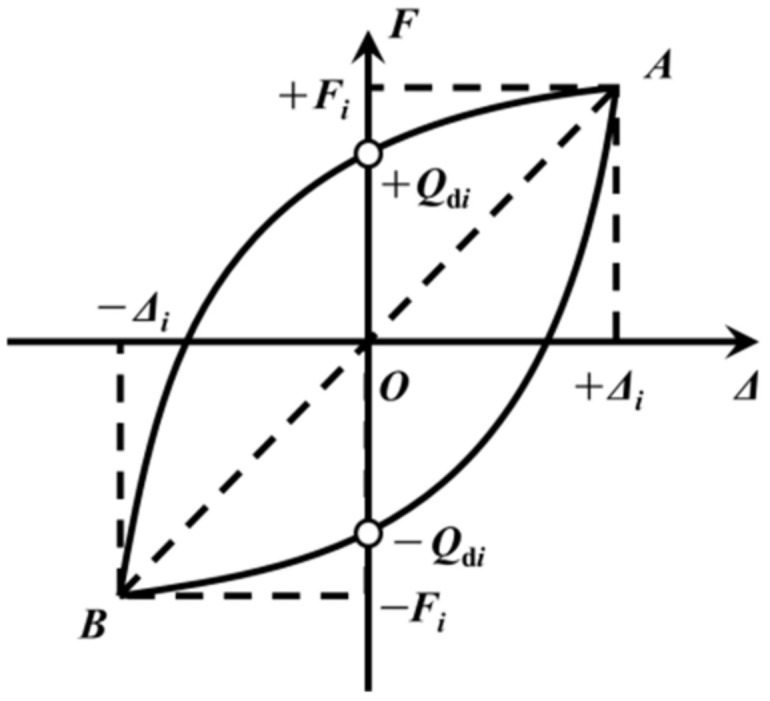
Restoring force model of the rubber bearing.

As per the specifications outlined in ‘Rubber bearing Part I: Test method of the rubber bearing’ (GB/T20688.1-2007) [35], the characteristic strength of a lead rubber bearing is determined by calculating the average absolute value of the *y*-axis intercept, as depicted in Figure 12.

(10) $$Q_{d,i}=\frac{1}{2}(\left|+Q_{di}\right|+\left|-Q_{di}\right|)$$

where $+Q_{di}$ is the positive intercept of the *i*-th hysteresis curve and the *y*-axis and $-Q_{di}$ is the negative intercept of the *i*-th hysteresis curve and *y*-axis, respectively.

**Figure 13:**
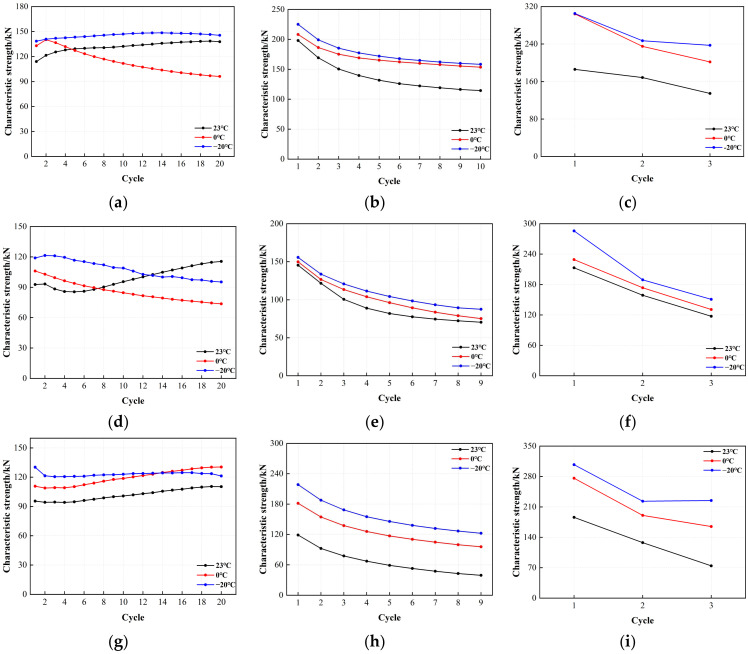
Variation of the characteristic strength with loading cycles for LRB900: (**a**) LRB900-1 (0.20 Hz-50%), (**b**) LRB900-1 (0.20 Hz-100%), (**c**) LRB900-1 (0.20 Hz-250%), (**d**) LRB900-2 (0.25 Hz-50%), (**e**) LRB900-2 (0.25 Hz-100%), (**f**) LRB900-2 (0.25 Hz-250%), (**g**) LRB900-3 (0.30 Hz-50%), (**h**) LRB900-3 (0.30 Hz-100%), and (**i**) LRB900-3 (0.30 Hz-250%).

The characteristic strength of the LRB900 bearings in each cycle was determined using the method described in the preceding section. Figure 13 illustrates the variation in characteristic strength with an increasing number of loading cycles. The characteristic strength of LRB900 displayed a notable response to work hardening, first decreasing at a moderate strain and then increasing with the accumulation of loading cycles under a 50% shear strain. The characteristic strength of LRB900 ranged from 13% to 26%, with no apparent correlation with temperature. In addition, when subjected to 100% and 250% shear strains, the characteristic strength of LRB900 decreased with the increasing number of loading cycles. The lead core underwent plastic deformation during the shear test, and its recrystallization behavior was significantly influenced by the temperature. When a shear strain of 100% was applied, the characteristic strength decreased from 33% to 53%. Similarly, when a shear strain of 250% was applied, the characteristic strength decreased by 27–45%. In addition, it is observed that the characteristic strength of LRB900 at −20 °C is significantly greater than at 0 °C and ambient temperature for both 100% and 250% shear strains. Additionally, there is a clear correlation between temperature and characteristic strength.

**Figure 14:**
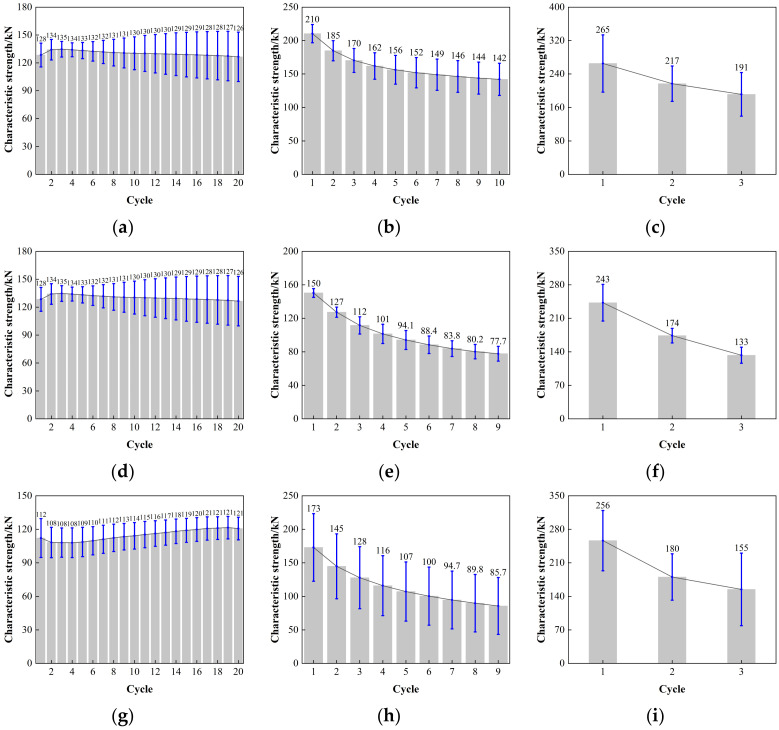
Comparison of the characteristic strength with loading cycles for LRB900: (**a**) LRB900-1 (0.20 Hz-50%), (**b**) LRB900-1 (0.20 Hz-100%), (**c**) LRB900-1 (0.20 Hz-250%), (**d**) LRB900-2 (0.25 Hz-50%), (**e**) LRB900-2 (0.25 Hz-100%), (**f**) LRB900-2 (0.25 Hz-250%), (**g**) LRB900-3 (0.30 Hz-50%), (**h**) LRB900-3 (0.30 Hz-100%), and (**i**) LRB900-3 (0.30 Hz-250%).

The error value analysis result for the characteristic strength of LRB900 is depicted in Figure 14. The error bars of the LRB900-1, LRB900-2, and LRB900-3 supports exhibit a relatively consistent length when subjected to shear strains of 50%, 100%, and 250%, respectively. These results indicate that the test data is quite stable, the discreteness is minimal, and the reliability is high.

#### 3.2.2. The Post-Yield Stiffness of LRB900

**Figure 15:**
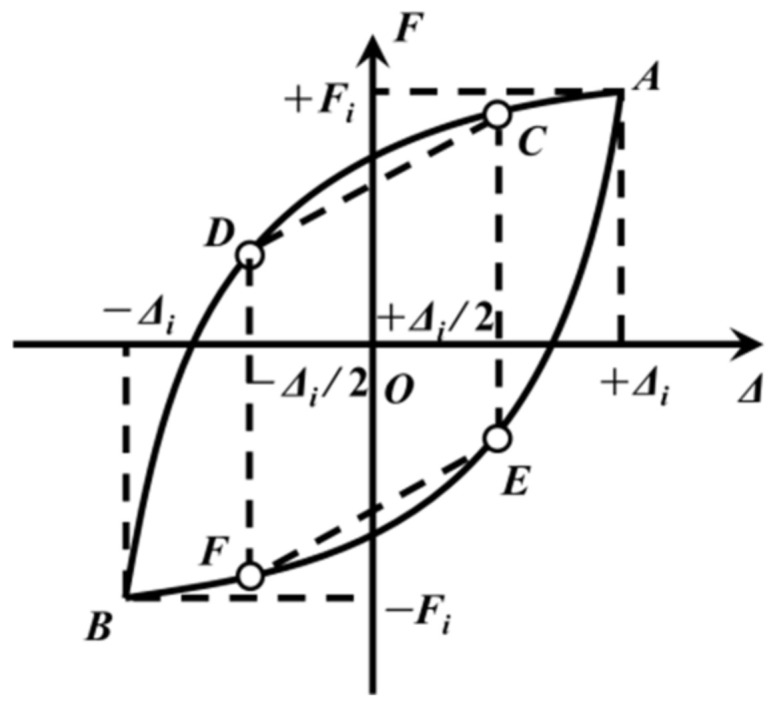
Definition of post-yield stiffness of the rubber bearing.

The post-yield stiffness of a lead–rubber bearing can be ascertained using the following equation:

(11) $$K_{post,i}=\frac{1}{2}(\frac{F_{C}-F_{D}}{X_{c}-X_{D}}+\frac{F_{E}-F_{F}}{X_{E}-X_{F}})$$

where *C*, *D*, *E*, and *F* are the four points at the displacement of Δ*_i_*/2 and −Δ*_i_*/2 on the hysteresis curve in Figure 15, and Δ*_i_* and −Δ*_i_* represent the maximum and minimum shear displacements in the *i*-th hysteresis loop, respectively. Consequently, the post-yield stiffness is therefore defined as the mean gradient of the *CD* and *EF* lines.

**Figure 16:**
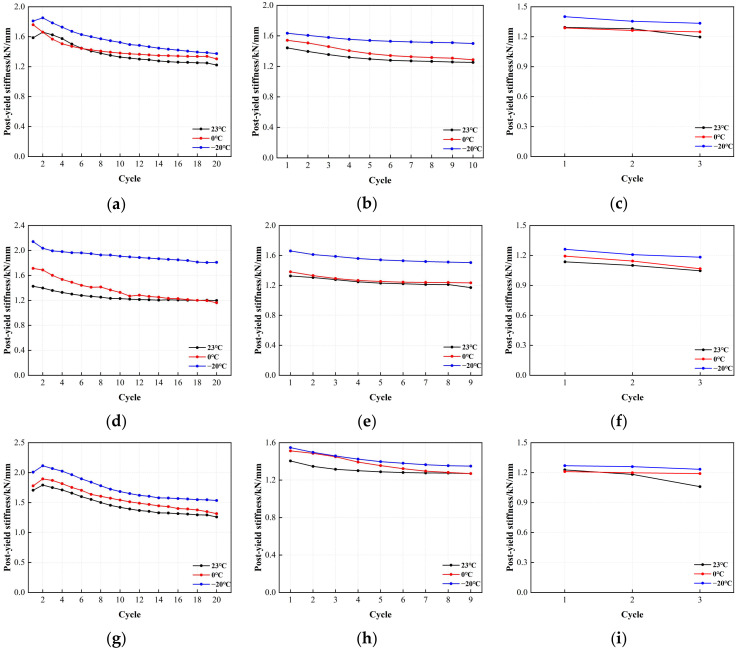
The variation curve of post-yield stiffness and loading cycles for LRB900: (**a**) LRB900-1 (0.20 Hz-50%), (**b**) LRB900-1 (0.20 Hz-100%), (**c**) LRB900-1 (0.20 Hz-250%), (**d**) LRB900-2 (0.25 Hz-50%), (**e**) LRB900-2 (0.25 Hz-100%), (**f**) LRB900-2 (0.25 Hz-250%), (**g**) LRB900-3 (0.30 Hz-50%), (**h**) LRB900-3 (0.30 Hz-100%), and (**i**) LRB900-3 (0.30 Hz-250%).

Figure 16 shows the post-yield stiffness of LRB900 as a function of the number of loading cycles. The post-yield stiffness showed a gradual and progressive decline under various shear stresses. The phenomenon is primarily attributable to the post-yield stiffness of the lead rubber bearing, which is primarily manifested in the mechanical properties of the rubber material. The post-yield stiffness of LRB900 decreases from 21% to 29% when subjected to a shear strain of 50%. Similarly, at a shear strain of 100%, the post-yield stiffness decreases by 10% to 13%. Ultimately, a shear strain of 250% resulted in a reduction in post-yield stiffness by 5–8%. Although there is a correlation between the post-yield stiffness and temperature, it is worth noting that the disparity among the three temperatures is insignificant.

**Figure 17:**
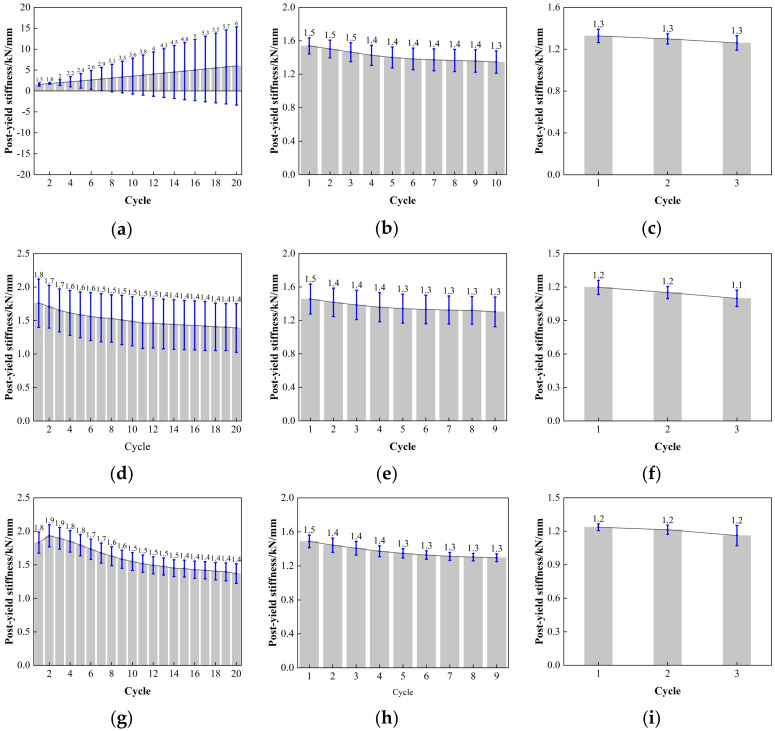
Comparison of the post-yield stiffness with loading cycles for LRB900: (**a**) LRB900-1 (0.20 Hz-50%), (**b**) LRB900-1 (0.20 Hz-100%), (**c**) LRB900-1 (0.20 Hz-250%), (**d**) LRB900-2 (0.25 Hz-50%), (**e**) LRB900-2 (0.25 Hz-100%), (**f**) LRB900-2 (0.25 Hz-250%), (**g**) LRB900-3 (0.30 Hz-50%), (**h**) LRB900-3 (0.30 Hz-100%), and (**i**) LRB900-3 (0.30 Hz-s250%).

The error value analysis result for the post-yield stiffness of LRB900 is depicted in Figure 17. Discreteness is observed in LRB900-1, LRB900-2, and LRB900-3 when the shear strain is 50%. The analysis reveals that when subjected to a minor strain of 50%, the work hardening of the lead core significantly impacts the characteristic strength of the bearing, indicating a degree of instability. The length of the error bar for LRB900-1, LRB900-2, and LRB900-3 bearings is comparatively consistent when subjected to 100% and 250% action, indicating a high level of credibility, stability, and discreteness in the test data.

#### 3.2.3. Horizontal Equivalent Stiffness of LRB900 and LNR900

The calculation for the horizontal equivalent stiffness of the rubber bearing is as follows:

(12) $$K_{h,i}=\frac{F_{i}-(-F_{i})}{\Delta_{i}-(-\Delta_{i})}$$

where *F_i_* and −*F_i_* are the restoring forces corresponding to the maximum and minimum displacements, respectively, in the *i*-th hysteresis loop of Figure 12.

**Figure 18:**
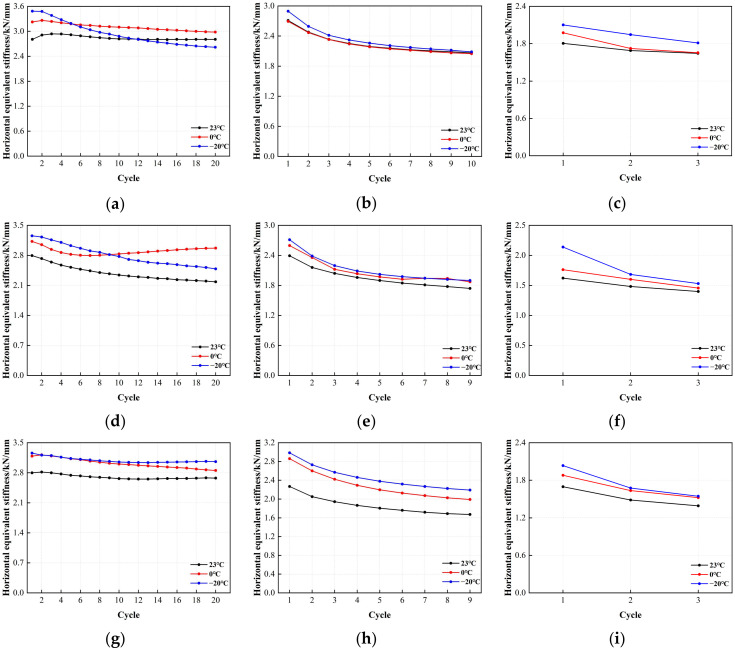
The variation curve of horizontal stiffness and loading cycles for LRB900: (**a**) LRB900-1 (0.20 Hz-50%), (**b**) LRB900-1 (0.20 Hz-100%), (**c**) LRB900-1 (0.20 Hz-250%), (**d**) LRB900-2 (0.25 Hz-50%), (**e**) LRB900-2 (0.25 Hz-100%), (**f**) LRB900-2 (0.25 Hz-250%), (**g**) LRB900-3 (0.30 Hz-50%), (**h**) LRB900-3 (0.30 Hz-100%), and (**i**) LRB900-3 (0.30 Hz-250%).

Figure 18 shows the correlation between the horizontal equivalent stiffness of LRB900 and the number of cyclic loading cycles. The horizontal equivalent stiffness of LRB900 varied with the increasing number of loading cycles, similar to its characteristic strength. (1) At 50% shear strain, the horizontal equivalent stiffness of LRB900 decreased from 8% to 19%, regardless of the temperature variations. (2) At shear strains of 100% and 250%, the characteristic strength of the bearing decreased with the increasing number of loading cycles, consequently leading to a reduction in horizontal equivalent stiffness. At a shear strain of 100%, the horizontal equivalent stiffness decreased from 25% to 28%. At a shear strain of 250%, the horizontal equivalent stiffness decreased by 13–20%. In addition, the horizontal equivalent stiffness demonstrated a noticeable temperature dependence under the conditions of 100% and 250% shear strain. Specifically, the horizontal equivalent stiffness increased with decreasing temperature. This behavior can be attributed to the significant increase in the load-carrying capacity of the lead core at lower temperatures.

**Figure 19:**
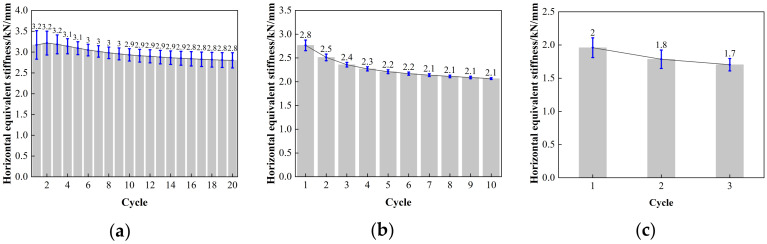

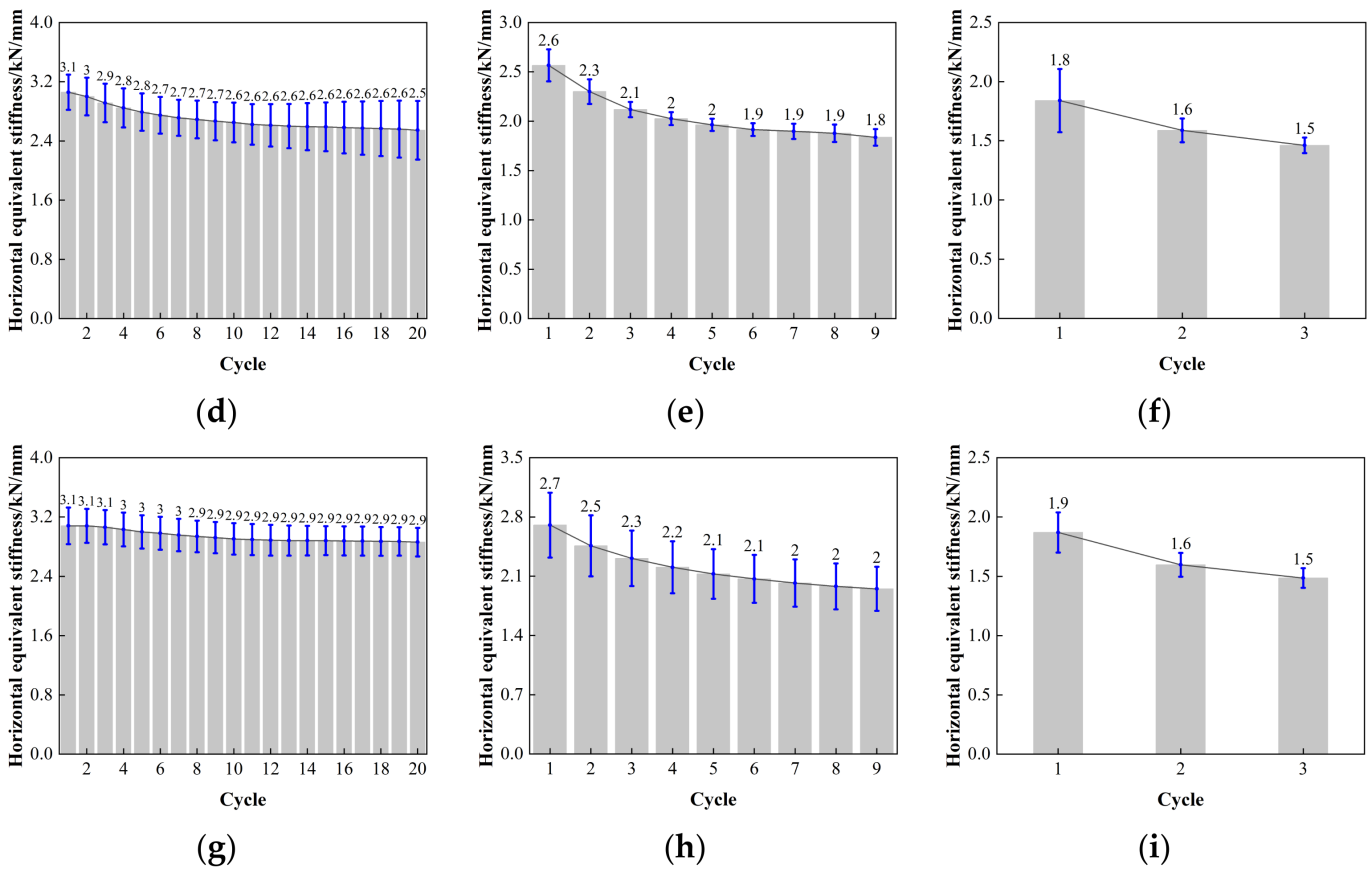
Comparison of the horizontal equivalent stiffness with loading cycles for LRB900: (**a**) LRB900-1 (0.20 Hz-50%), (**b**) LRB900-1 (0.20 Hz-100%), (**c**) LRB900-1 (0.20 Hz-250%), (**d**) LRB900-2 (0.25 Hz-50%), (**e**) LRB900-2 (0.25 Hz-100%), (**f**) LRB900-2 (0.25 Hz-250%), (**g**) LRB900-3 (0.30 Hz-50%), (**h**) LRB900-3 (0.30 Hz-100%), and (**i**) LRB900-3 (0.30 Hz-250%).

Figure 19 depicts the result of the error value analysis for the horizontal equivalent stiffness of LRB900. The error bar length of the LRB900-1, LRB900-2, and LRB900-3 supports is comparatively consistent at shear strains of 50%, 100%, and 250%. This indicates that the test data is relatively stable, with minimal dispersion, and that the reliability is high.

**Figure 20:**
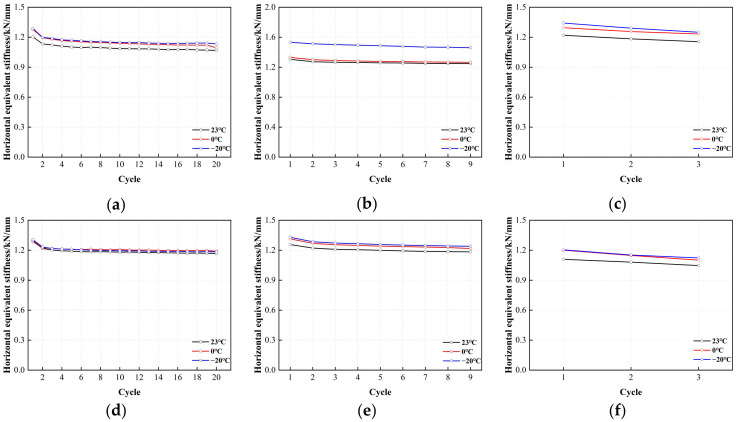

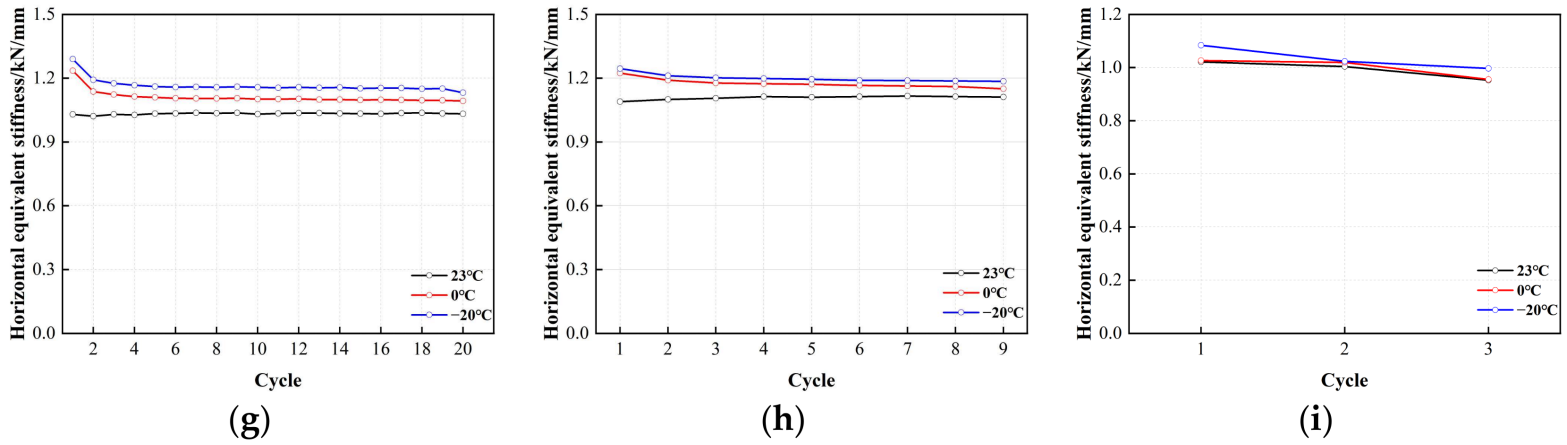
The variation curve of horizontal stiffness and loading cycles for LNR900: (**a**) LNR900-1 (0.20 Hz-50%), (**b**) LNR 900-1 (0.20 Hz-100%), (**c**) LNR900-1 (0.20 Hz-250%), (**d**) LNR900-2 (0.25 Hz-50%), (**e**) LNR900-2 (0.25 Hz-100%), (**f**) LNR900-2 (0.25 Hz-250%), (**g**) LNR900-3 (0.30 Hz-50%), (**h**) LNR900-3 (0.30 Hz-100%), and (**i**) LNR900-3 (0.30 Hz-250%).

The horizontal equivalent stiffness of LNR900, as depicted in Figure 20, exhibits a reasonably constant decrease in the post-yield horizontal stiffness of the lead rubber bearing. Although there is a correlation between the temperatures of −20 °C, 0 °C, and 23 °C, the disparity between them is minimal. At a minor strain of 50%, the horizontal equivalent stiffness of the LNR900 bearing decreased from 8–13%. The LNR900 bearing degraded by 4–7% under 100% shear strain, and a similar degradation range of 4–7% was observed under 250% shear strain.

**Figure 21:**
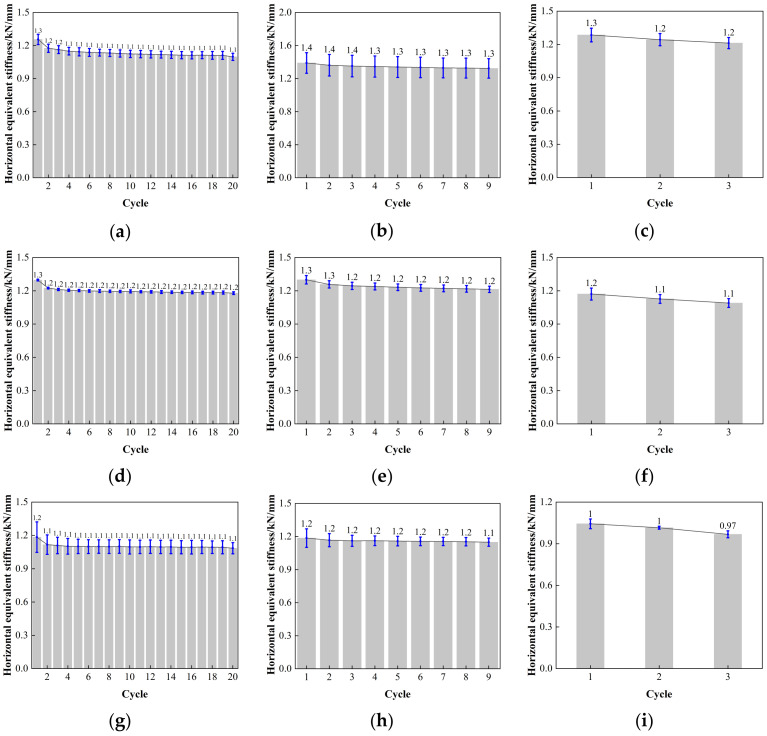
Comparison of the horizontal equivalent stiffness with loading cycles for LNR900: (**a**) LNR900-1 (0.20 Hz-50%), (**b**) LNR900-1 (0.20 Hz-100%), (**c**) LNR900-1 (0.20 Hz-250%), (**d**) LNR900-2 (0.25 Hz-50%), (**e**) LNR900-2 (0.25 Hz-100%), (**f**) LNR900-2 (0.25 Hz-250%), (**g**) LNR900-3 (0.30 Hz-50%), (**h**) LNR900-3 (0.30 Hz-100%), and (**i**) LNR900-3 (0.30 Hz-250%).

Figure 21 is the error value analysis result of LNR900 horizontal equivalent stiffness. When the shear strain is 50%, 100%, and 250%, the length of the error bar of LNR900-1, LNR900-2, and LNR900-3 supports is relatively uniform, the test data is relatively stable, the discreteness is small, and the reliability is high.

#### 3.2.4. The Equivalent Damping Ratio of LRB900

The calculation of the equivalent damping ratio can be obtained from the following equation:

(13) $$h=\frac{S_{APBQ}}{2\pi (S_{OAS}+S_{OBR})}$$

**Figure 22:**
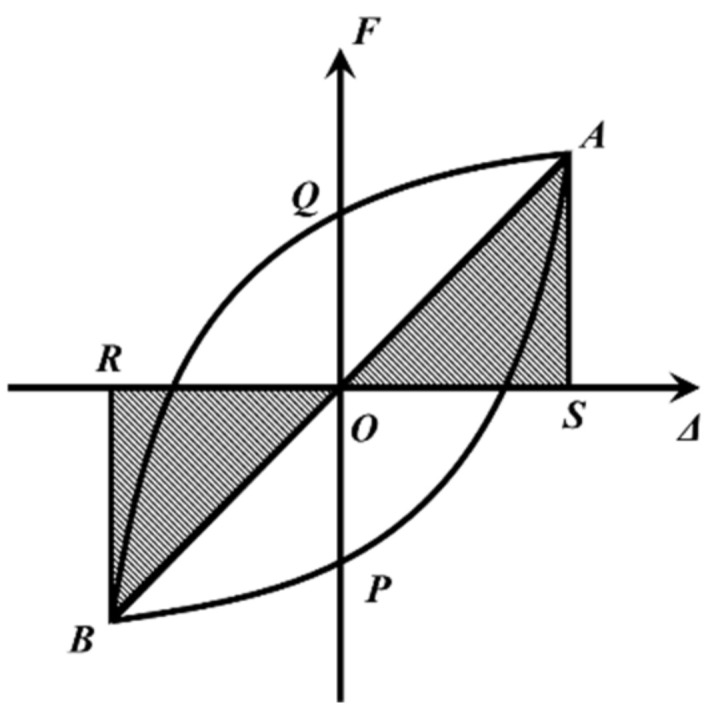
Definitions of the equivalent damping ratio.

The areas designated as *S_APBQ_*, *S_OAS_*, and *S_OBR_* are considered enclosed spaces in Figure 22.

**Figure 23:**
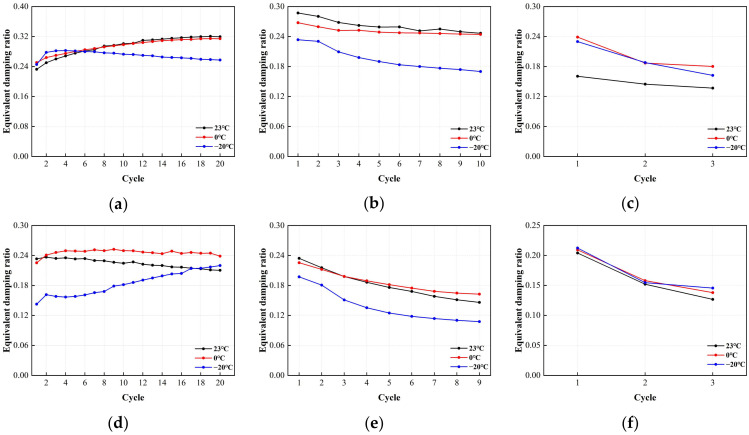

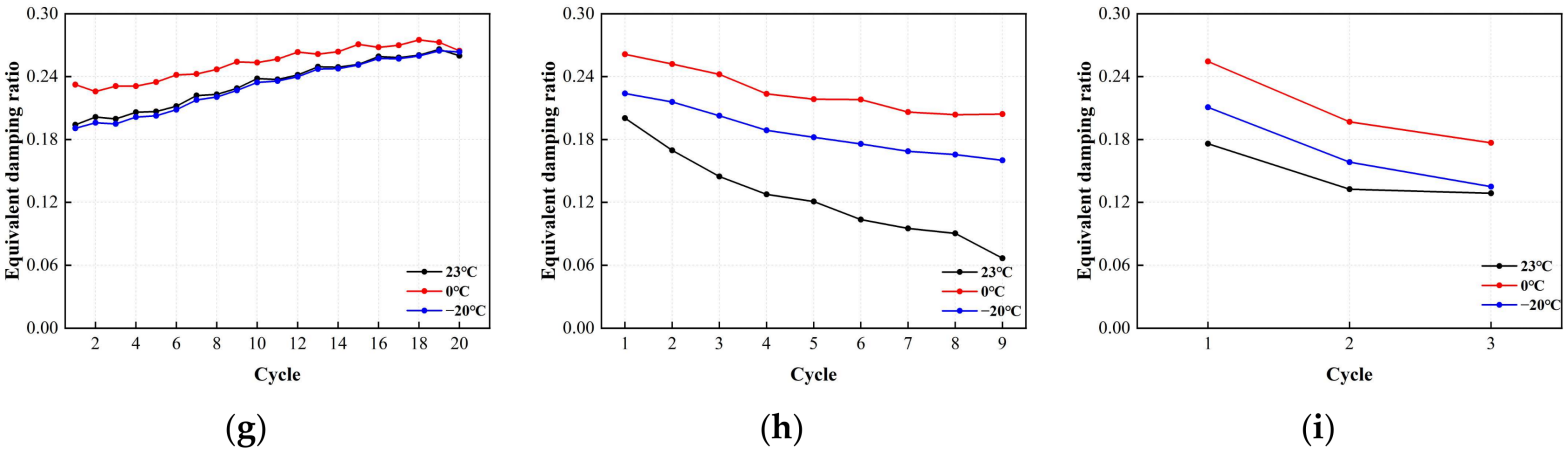
The variation curve of equivalent damping ratio and loading cycles for LRB900: (**a**) LRB900-1 (0.20 Hz-50%), (**b**) LRB900-1 (0.20 Hz-100%), (**c**) LRB900-1 (0.20 Hz-250%), (**d**) LRB900-2 (0.25 Hz-50%), (**e**) LRB900-2 (0.25 Hz-100%), (**f**) LRB900-2 (0.25 Hz-250%), (**g**) LRB900-3 (0.30 Hz-50%), (**h**) LRB900-3 (0.30 Hz-100%), and (**i**) LRB900-3 (0.30 Hz-250%).

The correlation between the equivalent damping ratio of LRB900 and the number of loading cycles is shown in Figure 23. According to the analysis, the equivalent damping ratio did not exhibit significant sensitivity to temperature variations across a range of shear strains. (1) Under the action of 50% small strain, the equivalent damping ratio of LRB900 fluctuates by 19–24%, and there is no significant temperature correlation observed. (2) Under 100% medium strain, the equivalent damping ratio of the support decreases by 17–39%, while under 250% high strain, the equivalent damping ratio of the support decreases by 23–35%. (3) At medium and large strains, the equivalent damping ratio of LRB900 gradually decreases due to the heating and softening effect of the lead core, indicating that the energy dissipation capacity of LRB900 bearings gradually decreases.

**Figure 24:**
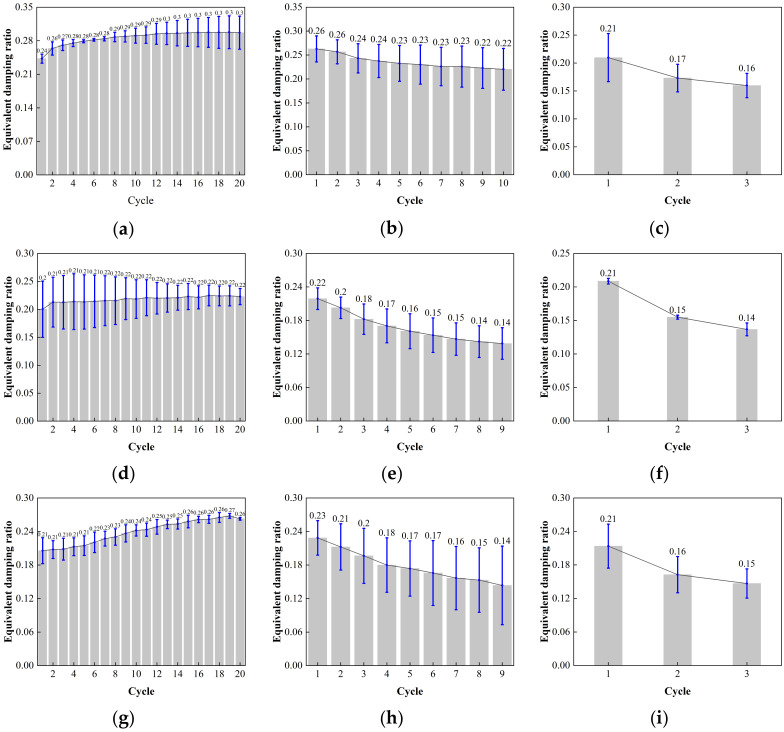
Comparison of the equivalent damping with loading cycles for LRB900: (**a**) LRB900-1 (0.20 Hz-50%), (**b**) LRB900-1 (0.20 Hz-100%), (**c**) LRB900-1 (0.20 Hz-250%), (**d**) LRB900-2 (0.25 Hz-50%), (**e**) LRB900-2 (0.25 Hz-100%), (**f**) LRB900-2 (0.25 Hz-250%), (**g**) LRB900-3 (0.30 Hz-50%), (**h**) LRB900-3 (0.30 Hz-100%), and (**i**) LRB900-3 (0.30 Hz-250%).

Figure 24 displays the error value analysis results for the LRB900 equivalent damping ratio. At shear strain levels of 50%, 100%, and 250%, the error bar lengths for LRB900-1, LRB900-2, and LRB900-3 supports are consistent. The test data shows stability, low discreteness, and good dependability.

### 3.3. The Adjustment Coefficient Considering the Influence of Ambient Temperature

Regarding the influence of ambient temperature on the mechanical properties of rubber bearings, three adjustment coefficients are given in Appendix D of the ‘Standard for Seismic Isolation Design of Building’ [36]. These coefficients are known as the adjustment coefficients for characteristic strength, post-yield stiffness, and horizontal equivalent stiffness, respectively.

(14) $$Q_{d}(T)=\frac{Q_{d}(T)}{Q_{d}(23\;℃)}Q_{d}(23\;℃)=C_{Q_{d}}(T)Q_{d}(23\;℃)$$

(15) $$K_{post}(T)=\frac{K_{post}(T)}{K_{post}(23\;℃)}K_{post}(23\;℃)=C_{K_{post}}(T)K_{post}(23\;℃)$$

(16) $$K_{h}=\frac{K_{h}(T)}{K_{h}(23\;℃)}K_{h}(23\;℃)=C_{K_{h}}(T)K_{h}(23\;℃)$$

To determine the three aforementioned adjustment coefficients, the present study used statistical analytical techniques to condense the hysteresis curves of LRB900 and LNR900. The first hysteresis curve was used to alleviate the influence of the hysteresis loading on the temperature of the lead core. Repetitive application of reverse stresses to the specimens was effective in mitigating the Scragging and Mullin effects. The adjustment coefficients for temperature sensitivity are calculated by dividing the test results obtained at 0 °C and −20 °C by the measured values at 23 °C.

The adjustment coefficient of characteristic strength and post-yield stiffness with the LRB900 can be expressed respectively as follows:

(17) $$C_{Q_{d}}(T)=1.2140e^{-0.0075T}$$

(18) $$C_{K_{post}}(T)=0.1261e^{-0.0306T}+0.9376$$

The adjustment coefficient of horizontal equivalent stiffness with the LRB900 and LNR900 can be also expressed respectively as follows:

(19) $$C_{K_{h}}(T)=1.1060e^{-0.0042T}$$

(20) $$C_{K_{h}}(T)=1.0590e^{-0.0023T}$$

where $C_{Q_{d}}\;\left(T\right)$ is the adjustment coefficient of the characteristic strength, $C_{K_{post}}\;\left(T\right)$ is the adjustment coefficient of the post-yield stiffness, and $C_{K_{h}}\;\left(T\right)$ is the adjustment coefficient of the horizontal equivalent stiffness.

**Figure 25:**
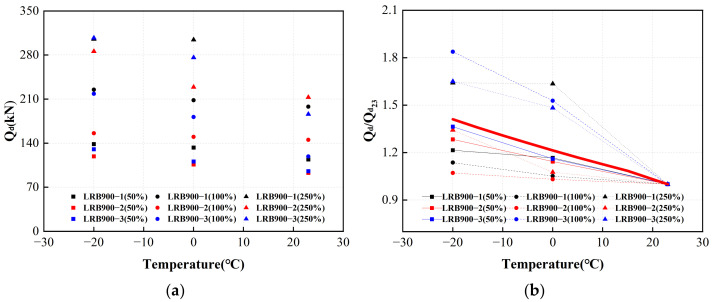
The adjustment coefficient of the characteristic strength of LRB900 considering the influence of ambient temperature: (**a**) The characteristic strength of LRB900 in the first hysteretic loop; (**b**) The characteristic strength temperature correction fitting curve of LRB900.

The characteristic strengths of the first hysteresis loops of LRB900 under various loading conditions are shown in Figure 25a. The red line in Figure 25b represents the adjustment coefficient curve after normalization. The characteristic strength of LRB900 increased by 21% at 0 °C compared to that at 23 °C. At a temperature of −20 °C, the characteristic strength of LRB900 increases by 41% compared to 23 °C.

From the above analysis, it is clear that the characteristic strength of isolation rubber bearings is underestimated at low temperatures, delaying the time when the isolation layer enters the elastic-plastic stage for energy consumption. This results in a decrease in the period of the isolation structure, a decrease in its isolation effect, and an increase in the risk of damage, collapse, and failure of the isolation structure.

**Figure 26:**
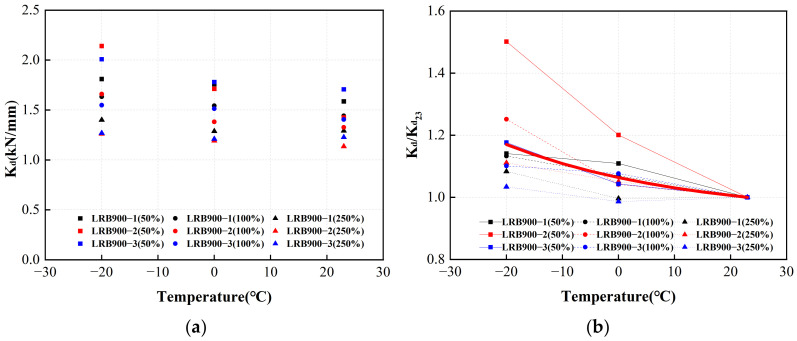
The adjustment coefficient of the post-yield stiffness of LRB900 considering the influence of ambient temperature: (**a**) The post-yield stiffness of LRB900 in the first hysteretic loop; (**b**) The post-yield stiffness temperature correction fitting curve of LRB900.

Figure 26a shows the post-yield stiffness of LRB900 during the initial loading cycle under varying conditions. The red line in Figure 26b represents the adjustment coefficient curve. At an ambient temperature of 0 °C, it can be seen that the post-yield stiffness of LRB900 is 6% higher than that at 23 °C. At an ambient temperature of −20 °C, the post-yield stiffness of LRB900 exhibits a 17% increase compared to the stiffness observed at 23 °C.

**Figure 27:**
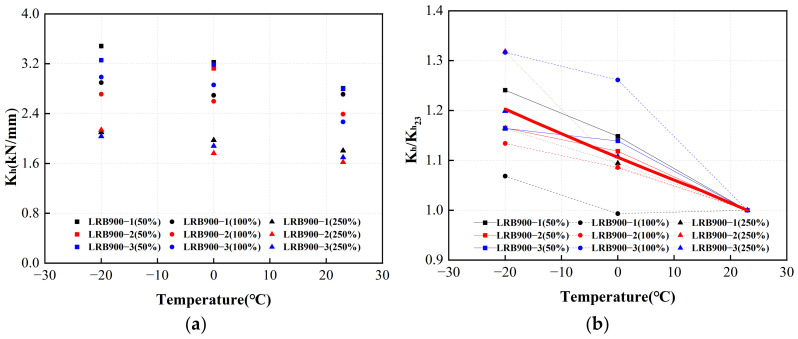
The adjustment coefficient of the horizontal equivalent stiffness of LRB900 considering the influence of ambient temperature: (**a**) The horizontal equivalent stiffness of LRB900 in the first hysteretic loop; (**b**) The horizontal equivalent stiffness temperature correction fitting curve of LRB900.

**Figure 28:**
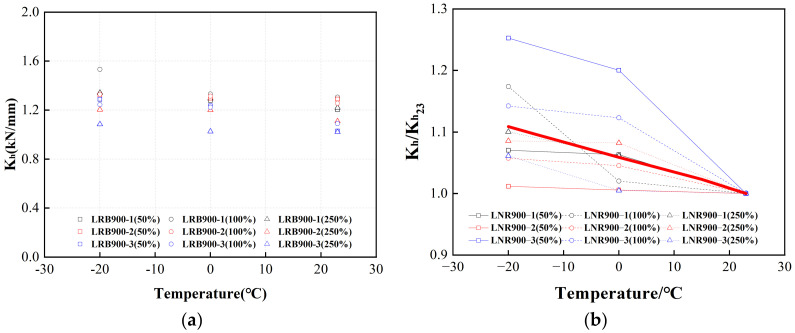
The adjustment coefficient of the horizontal equivalent stiffness of LNR900 considering the influence of ambient temperature: (**a**) The horizontal equivalent stiffness of LNR900 in the first hysteretic loop; (**b**) The horizontal equivalent stiffness temperature correction fitting curve of LNR900.

Figure 27a and Figure 28a illustrate the horizontal equivalent stiffnesses of LRB900 and LNR900 during the initial cycle, considering various loading situations. The red lines in Figure 27b and Figure 28b represent the adjustment coefficient curves. In contrast to the ambient temperature of 23 °C, the horizontal equivalent stiffness of LRB900 and LNR900 increased by 11% and 6%, respectively, when subjected to an ambient temperature of 0 °C. At a temperature of −20 °C, there was an increase of 20% and 11% for the corresponding variables.

From the above analysis, it can be seen that at low temperatures, the horizontal equivalent stiffness of the lead rubber bearings is underestimated, resulting in a decrease in the period of the isolation structure, an increase in the seismic load borne by the isolation structure, and an increase in the risk of failure of the upper structure.

The research undertaken in this study focused on investigating the temperature correlation tests within the confines of the LRB900 and LNR900 bearing types as well as a certain range of strain amplitudes. Future research will seek to broaden the range of ambient-temperature test conditions, thereby expanding the comprehensiveness of the experimental database. During the interim period, it is crucial to construct a finite element model for a rubber bearing that can effectively predict its mechanical properties. This model must account for the effects of ambient temperature and hysteresis heat. To attain an optimal theoretical framework, suitable adjustment coefficients must be incorporated into the performance index during the development of an isolated structure.

## 4. Conclusions

In this study, a sequence of high-speed compression-shear experiments were performed on seismic rubber bearings with a diameter of 900 mm. The objective was to investigate the effects of the loading frequency, shear strain amplitude, and ambient temperature. In addition, a correction approach is proposed to account for the internal inertial and frictional forces present within the loading system. This correction method aims to precisely ascertain the mechanical performance indices of the specimens. The analysis presented in this paper focuses on the mechanical performance indices of LRB900 and LNR900, paying special attention to their responses under varying loading conditions, as indicated by the revised experimental data. Finally, to integrate the influence of ambient temperature into the process of designing seismic structures, a corresponding correction coefficient is provided in accordance with the pertinent regulations. Therefore, the following conclusions were drawn:

(1) During high-speed and high-pressure loading tests, the friction or inertia force of the system is greater than 1% of the recorded shear force, therefore, it is necessary to correct the inertial force and shear force. The corrective method demonstrated in this study effectively mitigates the negative effects caused by the inertia of moving components and internal system friction.

(2) The observed trends for LRB900 were associated with an increase in the number of loading cycles. When subjected to a shear strain of 50%, the characteristic strength exhibited an initial decline, followed by an increase. Subsequently, it decreased from 33% to 53% at a shear strain of 100% and from 27% to 45% at a shear strain of 250%. The post-yield stiffness gradually decreased at various shear strains, with gradual declines of 21–29% at 50% shear strain, 10–13% at 100% shear strain, and 5–8% at 250% shear strain. Furthermore, the horizontal equivalent stiffness decreased by 8–19% at 50% shear strain, 25–28% at 100% shear strain, and 13–20% at 250% large strain. The horizontal equivalent stiffness of LNR900 decreased from 8% to 13% when subjected to a minor strain of 50%. Similarly, at shear strains of 100% and 250%, the stiffness decreased from 4% to 7%. The equivalent damping ratio shows fluctuations between 19% and 24% at a shear strain of 50%, whereas it decreases by 17% to 39% at a shear strain of 100% and falls by 23% to 35% at a shear strain of 250%. Insights into the interconnections among characteristic strength, post-yield stiffness, horizontal equivalent stiffness, and temperature emerged from the test data: as the number of loading cycles increased, these relationships became more evident. Nevertheless, the relationship between the temperature and the equivalent attenuation ratio is not readily apparent.

(3) The mechanical performances of LRB900 and LNR900 were significantly influenced by ambient temperature. Specifically, at a temperature of 0 °C, the characteristic strength of LRB900 increases by 21%, and its post-yield stiffness increases by 6% compared to a temperature of 23 °C. Moreover, both LRB900 and LNR900 experience an 11% increase in horizontal equivalent stiffness at 0 °C. Similarly, at a temperature of −20 °C, the characteristic strength and post-yield stiffness of LRB900 increase by 41% and 17%, respectively, compared to a temperature of 23 °C. Moreover, both LRB900 and LNR900 demonstrate a 20% increase in horizontal equivalent stiffness at −20 °C.

## Figures and Tables

**Table 1:** The dimensions of the specimens.

Main Parameter	Unit	LRB900	LNR900
Diameter of the bearing	mm	900	900
Lead diameter	mm	131.400	-
Diameter of the cover plate	mm	1200	1200
Thickness of the rubber sheet	mm	7.125	7.125
Number of the rubber sheet	layer	24	24
Thickness of the steel shim	mm	4	4
Total thickness of the rubber	mm	23	23
Height of the bearing	mm	92	92

**Table 2:** LRB900 (LNR900) test cases.

Bearing Type	Vertical Pressure (MPa)	Vertical Force (kN)	Loading Frequency (Hz)	Shear Strain	Horizontal Displacement (mm)	Peak Velocity (mm/s)
LRB900 (LNR900)	15	9540	0.20	50%	85.5	107.4
				100%	171	214.9
				250%	427.5	537.2
			0.25	50%	85.5	134.3
				100%	171	268.6
				250%	427.5	671.5
			0.30	50%	85.5	161.2
				100%	171	322.3
				250%	427.5	805.8

**Table 3:** Verification of the accuracy of equipment inertia force and friction force correction methods.

Bearing Type	Specimen Number	Characteristic Strength (kN)		Differentials %	Post-Yield Stiffness (kN/mm)		Differentials %
		Factory Inspection Results	Official Test Results		Factory Inspection Results	Official Test Results	
LRB900-50%	1 (0.20 Hz)	120.1121	110.5031	8	1.8435	1.7329	6
	2 (0.25 Hz)	114.8399	111.3947	3	1.8450	1.8081	2
	3 (0.30 Hz)	113.0938	106.3081	6	2.1641	2.1208	2
LRB900-100%	1 (0.20 Hz)	133.0646	130.4033	2	1.5162	1.4707	3
	2 (0.25 Hz)	131.1803	121.9976	7	1.5325	1.4405	6
	3 (0.30 Hz)	128.1337	121.7271	5	1.7664	1.6781	5
LRB900-250%	1 (0.20 Hz)	202.6552	190.4959	6	1.3439	1.2767	5
	2 (0.25 Hz)	165.0360	158.4346	4	1.1931	1.1693	2
	3 (0.30 Hz)	169.0976	158.9518	6	1.4097	1.3674	3

## Data Availability

Data are contained within the article.
